# Framing the Convergence of One Health and Digital Health in the Global South With a Gender-Sensitive Foresight Perspective: Delphi Study Using Latent Semantic Analysis

**DOI:** 10.2196/78702

**Published:** 2026-02-18

**Authors:** Jude Kong, Nicola Luigi Bragazzi

**Affiliations:** 1 Artificial Intelligence & Mathematical Modeling Lab (AIMMLab) Dalla Lana School of Public Health University of Toronto Toronto, ON Canada; 2 Institute of Health Policy Management and Evaluation (IHPME) University of Toronto Toronto, ON Canada; 3 Department of Mathematics Bahen Centre for Information Technology University of Toronto Toronto, ON Canada; 4 Africa-Canada Artificial Intelligence and Data Innovation Consortium (ACADIC) Toronto, ON Canada; 5 Global South Artificial Intelligence for Pandemic and Epidemic Preparedness and Response Network (AI4PEP) Toronto, ON Canada

**Keywords:** Delphi method, digital health, emerging issues, foresight, gender, Global South, latent semantic analysis, One Health, structural drivers, weak signals

## Abstract

**Background:**

The convergence of digital health and One Health represents an emergent paradigm in global health governance. While widely discussed in high-income settings, there is limited understanding of how this convergence is conceptualized in the Global South, particularly when viewed through a gender- and equity-sensitive foresight lens.

**Objective:**

This study aimed to map and classify expert discourse on digital health, One Health, and their convergence in the Global South using latent semantic analysis, with particular attention to structural drivers, emerging issues, weak signals, and gendered patterns of anticipation.

**Methods:**

A 3-round online Delphi survey was conducted with 45 experts from 19 countries across the Global South. Open-ended responses were analyzed using latent semantic analysis and stratified by gender. A foresight framework was applied to categorize topics as structural drivers, emerging issues, or weak signals, based on their temporal persistence, salience, and consensus.

**Results:**

In digital health, structural drivers included the systemic integration of digital technologies into public health systems, strategic alignment, and infrastructure development. Emerging issues comprised the adoption of artificial intelligence, chronic disease management via mobile health, and concerns about digital inclusion and interoperability. Weak signals included feminist digital ethics, trust in digital systems, and relational accountability—more frequently emphasized by female experts. In One Health, structural drivers were centered on intersectoral coordination, ecological integration, and the institutionalization of health-environment frameworks. Emerging issues encompassed anticipatory risk governance, food system sustainability, and the integration of environmental and population-level data. Weak signals included indigenous knowledge systems, subnational antimicrobial resistance governance, and structural underinvestment in ecological public health, with gendered divergence in framing. In the convergence discourse (digital health and One Health), structural drivers focused on the integration of digital surveillance systems, data infrastructures, and health information platforms to operationalize One Health. Emerging issues included climate-triggered system redesign, artificial intelligence and ecological monitoring, and the governance of cross-sectoral data. Weak signals pointed to algorithmic bias in zoonotic prediction, digital sovereignty in environmental health, and feminist critiques of convergence—all thematically rich but peripheral in consensus.

**Conclusions:**

This study revealed a multilayered and gender-influenced foresight architecture shaping the future of digital health and One Health in the Global South. Structural drivers denote maturing domains of implementation, while emerging issues and weak signals highlight latent, often overlooked opportunities and tensions. Incorporating equity-sensitive and gender-aware foresight methods is essential for crafting inclusive and anticipatory health governance strategies.

## Introduction

In an increasingly interconnected and rapidly changing world, the convergence of demographic aging, epidemiological transitions, and climate-related environmental threats has contributed to a multifaceted global health burden, placing immense strain on already fragile health care systems [1,2]. This burden is especially acute in the Global South, where both communicable and noncommunicable diseases persist amid structural inequalities and constrained resources [3]. Simultaneously, growing awareness of the complex, nonlinear, and interacting determinants of health has spurred a paradigmatic shift in the governance of health—away from siloed, biomedical models toward more holistic, systemic, and integrative frameworks [4].

The One Health paradigm, which acknowledges the interdependence of human, animal, and environmental health, has emerged as a critical response to zoonotic threats, ecosystem degradation, and planetary instability [5,6]. In parallel, the digital transformation of health—encompassed within the digital health agenda—has accelerated, leveraging data-driven technologies to enhance diagnostics, disease surveillance, and care delivery [7]. Their convergence, conceptualized as Digital One Health or One Digital Health, reflects an emergent field characterized by interdisciplinary cooperation and technologically mediated multisectoral coordination [8,9]. However, existing scholarship has largely framed such convergence through a Global North lens, overlooking the digital disparities, infrastructural deficits, and sociotechnological asymmetries that define the Global South [10]. Specifically, studies conducted in high-income and Global North settings have shown that the convergence of digital health and One Health is primarily operationalized through integrated surveillance systems, advanced health and environmental data infrastructures, and artificial intelligence (AI)–enabled early warning platforms, with reported gains in outbreak detection, antimicrobial resistance (AMR) monitoring, and cross-sectoral coordination. These contributions, while methodologically and technologically robust, are predominantly grounded in contexts characterized by mature digital ecosystems, stable governance arrangements, and well-resourced regulatory frameworks, thereby limiting their transferability to the structural, institutional, and equity-related realities of the Global South [8-10].

Moreover, a gender-sensitive or feminist lens is often lacking, thereby marginalizing the situated experiences, epistemologies, and agency of women and gender-diverse actors in shaping health and technology ecosystems [10].

To address these epistemic and representational gaps, this study used a foresight-oriented text mining approach grounded in horizon scanning and the identification of weak signals, emerging issues, and structural drivers [10]. The goal was to map and classify the thematic architecture of the discourse on the convergence of One Health and digital health from a Global South perspective, with particular attention to gendered dynamics, equity-informed framings, and the coproduction of inclusive and pluralistic alternative futures.

## Methods

### Ethical Considerations

The study protocol was approved by the institutional review board of Dalla Lana School of Public Health, University of Toronto, Ontario, Canada (48350). The study was conducted in accordance with the Declaration of Helsinki, and written informed consent was obtained from all participants.

### Expert Recruitment

Experts were recruited through a purposive sampling strategy aimed at capturing diverse perspectives on the convergence of digital health and One Health within the Global South. Invitations were disseminated via professional networks, relevant mailing lists, and institutional affiliations, targeting stakeholders actively engaged in health policy, digital innovation, epidemiology, veterinary medicine, environmental science, and global clinical public health practice.

Selection criteria included demonstrated expertise in at least 1 relevant domain, professional affiliation with an institution based in the Global South, and availability to complete multiple rounds of an online Delphi survey. A total of 45 experts completed all survey rounds (n=3) and were included in the final analysis. The Delphi process was conducted anonymously to encourage candid responses and reduce social desirability bias.

### Survey Instrument

The study used a structured, multiround Delphi survey designed to elicit expert consensus on the convergence of digital health and One Health in the Global South. The instrument was developed to capture both sociodemographic diversity and domain-specific insights relevant to foresight-oriented analysis. The survey instrument was developed ad hoc for the purposes of this study, as no validated or standardized questionnaire currently exists to capture expert foresight on the convergence of digital health and One Health, particularly from a Global South and gender-sensitive perspective.

Participants first provided background information, including age, gender, country of birth, country/countries of study, current residence, professional role, areas of expertise, and years of experience in digital health and/or One Health. They were also asked to reflect on the interdisciplinary nature of their work environments.

Each round of the Delphi survey included open-ended prompts focused on present and future opportunities, challenges, and disruptors in both digital health and One Health domains. Specific items addressed strategic drivers, structural barriers, project involvement, and anticipated synergies between the two fields. After each round, responses were thematically analyzed, and aggregated feedback was provided to participants in subsequent rounds, allowing for reflection, refinement, and convergence of expert opinion. The final item in each round requested participants to generate an anonymous identification code to enable matching across rounds. The survey was administered via Google Forms, ensuring broad accessibility across diverse geographies. All items were open-ended to enable narrative elaboration and thematic emergence, aligning with the exploratory aims of the foresight exercise.

The full set of survey items is provided in Multimedia Appendix 1.

### Overview of the Methodology: Text Mining and Foresight Exercise

To explore temporal dynamics and strategic orientations in stakeholder discourse on digital health and One Health in the Global South, we used comparative latent semantic analysis (LSA) [11,12] across different foresight-relevant prompts: (1) present implementation, (2) perceived opportunities, (3) perceived challenges, and (4) future-oriented opportunities or challenges. Each response set was subjected to LSA to extract latent topics, with eigenvalues and variance explained used to assess topic salience.

To interpret LSA outputs through a foresight lens, we used a 3-tiered classification framework widely used in foresight and futures research: structural drivers, emerging issues, and weak signals [13], enabling differentiation between deeply embedded system forces, nascent developments gaining stakeholder traction, and low-frequency yet potentially disruptive cues.

Structural drivers were defined as topics that (1) appeared across both present- and future-oriented datasets and (2) accounted for a substantial proportion of explained variance. These themes represent widely shared, resilient priorities. Emerging issues were operationalized as topics observed predominantly or exclusively in future-oriented discourse, with a moderate share of explained variance. These topics signal directional shifts in collective attention and can anticipate inflection points in system trajectories. Weak signals were identified as topics with low variance explained, often surfacing in only 1 temporal frame (present or future) and lacking broader consensus. Although marginal, these signals are analytically significant, as they may indicate overlooked risks, latent needs, or early-stage innovations that could reshape the field.

A gendered lens was applied throughout this classification to assess how male and female respondents may differentially emphasize structural versus emergent priorities. Gender-divergent weak signals, in particular, offer critical insight into situated epistemologies—highlighting perspectives that are often sidelined in dominant discourses.

Overall, this classification supports a foresight-informed interpretation of the LSA results and strengthens the capacity to generate equity-sensitive, gender-responsive scenarios grounded in both continuity and systemic change.

### Data Collection and Preprocessing

The dataset comprised the open-ended textual responses provided by the expert panel, compiled into a structured corpus for subsequent text mining. The entries varied in both length and complexity, reflecting the heterogeneous expertise and regional diversity of participants addressing digital health and One Health issues. Prior to computational analysis, the corpus underwent standard preprocessing procedures aimed at improving analytical robustness. Text normalization steps included tokenization, lowercasing, stop-word removal, punctuation stripping, and the elimination of nonalphabetical characters. Stemming and lemmatization were applied to reduce lexical variation while preserving semantic meaning. Importantly, domain-specific terminology was preserved throughout the process to maintain contextual integrity. Preprocessing was implemented using English-language stop word lists and stemming algorithms.

### Text Mining Analysis

LSA [11,12] was used to extract conceptual structures embedded within the textual data and to systematically identify latent themes in stakeholder discourse. As a well-established natural language processing technique, LSA enables the reduction of linguistic dimensionality by analyzing patterns of word cooccurrence, making it particularly suitable for foresight-driven text mining applications. A term-document matrix was constructed using a bag-of-words representation, incorporating a minimum term frequency threshold of 2 and a sparsity cutoff of 0.975. Dimensionality reduction was performed using singular value decomposition, which facilitated the identification of principal semantic axes and coherent topic clusters. Specifically, LSA decomposes the term-document matrix into 3 matrices (U, Σ, V^T^) via singular value decomposition, where Σ is a diagonal matrix containing the singular values associated with each latent semantic dimension. The squared singular values correspond to the eigenvalues of the latent dimensions and quantify the amount of semantic variance captured by each extracted topic. Eigenvalues were computed and ranked in descending order, without the application of predefined cutoffs or dimensionality reduction thresholds. In this analytical context, eigenvalues represent the relative semantic weight or salience of each latent topic, reflecting the strength and coherence of word cooccurrence patterns across expert responses rather than statistical significance in an inferential sense. Topics associated with higher eigenvalues correspond to dominant and widely shared thematic structures within the discourse, whereas topics associated with lower eigenvalues represent less frequent, more weakly articulated, or emerging semantic patterns. For each topic, the percentage of explained variance was calculated as the ratio between the eigenvalue of that topic and the sum of all retained eigenvalues, multiplied by 100. Explained variance therefore expresses the proportion of total semantic variance in the corpus accounted for by each latent dimension. This measure was used to assess thematic prominence and consensus across responses, rather than to perform hypothesis testing or probabilistic inference. Eigenvalues and explained variance were interpreted within a foresight-oriented analytical framework. Topics with consistently high explained variance across both present- and future-oriented prompts were classified as structural drivers, reflecting stable and deeply embedded priorities. Topics with moderate explained variance that emerged primarily in future-oriented discourse were classified as emerging issues, indicating evolving areas of collective attention. Topics characterized by low explained variance, limited consensus, or temporal specificity were classified as weak signals. Importantly, low explained variance was not interpreted as analytical irrelevance; rather, in line with horizon-scanning and futures methodologies, such topics were considered potentially indicative of early-stage, underexplored, or disruptive dynamics that may gain relevance over time. This interpretation strategy ensured conceptual alignment between the quantitative outputs generated by LSA and the qualitative, anticipatory objectives of the foresight exercise, while enabling transparent interpretation of the eigenvalues and explained variance.

LSA was first conducted on the overall corpus to extract global semantic patterns. Additional stratified analyses were performed based on participant gender. Following decomposition, the resulting topics were qualitatively interpreted and labeled by 2 independent coders (JK and NLB), who reviewed the most salient terms within each topic vector. Intercoder reliability was ensured through an iterative reconciliation process, with final topic labels established via consensus.

All LSA procedures were executed using the XLSTAT software suite (Lumivero), a commercial statistical package supporting advanced text analytics.

## Results

### Sample

The expert sample had a mean age of 43.1 (SD 10.3) years, reflecting midcareer professionals, and a male-to-female ratio of 2.8:1 (n=33, 73% men and n=12, 27% women). Geographically, the sample spanned 19 countries, predominantly in Africa (n=26, 58% participants), followed by Asia (n=14, 31% participants), Latin America (n=3, 7% participants), and the Middle East and North Africa region (n=2, 4% participants). Among female participants, 50% (6/12) were from Africa, 33.3% (4/12) from Asia, 8.3% (1/12) from Latin America, and 8.3% (1/12) from the Middle East and North Africa region. The distribution of participants by gender and geographic region did not differ significantly (Fisher exact test *P*=.77), indicating that female participants were not disproportionately concentrated in any single region despite their smaller overall number. Participants had a mean professional experience of 7.3 (SD 5.9) years, and represented diverse roles across academia, policy, investment, and practice, including directors, principal investigators, professors, consultants, and World Health Organization officers. Their professional involvement encompassed a wide array of digital health and One Health initiatives in the Global South, such as AI-driven disease prediction, electronic health records, mobile health (mHealth) applications, epidemic surveillance, and AMR monitoring in wastewater. Notably, many participants contributed to public health training, policy development, and the integration of emerging technologies to address syndemics at the human-animal-environment interface.

### Digital Health in the Global South

LSA applied to responses regarding the successful implementation of digital health in the Global South revealed 10 interpretable topics. Topic 1, explaining 46.75% of the variance, reflected structural and systemic priorities—particularly integration, infrastructure, and strategic alignment. Topic 2 (7.57%) captured operational themes around patient management and service delivery, while Topic 3 (4.77%) emphasized stakeholder engagement and community outreach. Topic 4 (4.21%) focused on governance and regulation, complemented by Topic 5 (4.04%) which stressed regulatory clarity and local adaptation. Topic 6 (3.57%) addressed digital literacy and technological access, and Topic 7 (2.99%) emphasized co-design and participatory processes. Topics 8-10, collectively explaining another 6.91%, covered scalability and sustainability (2.63%), capacity building (2.21%), and context-aware implementation frameworks (2.07%), respectively. Adopting a gender lens, common themes included strategic alignment (male Topic 7: 2.68%; female Topic 6: 1.56%), capacity building (male Topic 5: 3.90%; female Topic 3: 9.22%), and patient-centered approaches (male Topic 2: 8.53%; female Topic 9: 0.83%), though to a different degree. Both groups also addressed community and infrastructure support, with the theme being more prominent in women—Topic 1 (52.95%) versus Topic 8 (2.35%). Male responses uniquely stressed technical and structural aspects, such as the importance of system-wide deployment and digital solution rollouts (Topic 1: 50.18%), project-level security and awareness (Topic 6: 3.37%), and digital health literacy (Topic 4: 4.53%), as well as affordability and device regulation (Topic 3: 5.10%), financial sustainability (Topic 10: 2.11%), and framework scaling (Topic 9: 2.22%). Female respondents, in contrast, highlighted intersectoral dimensions, with attention to collaboration among stakeholders and financial dependencies (Topic 4: 3.74%) and multiactor coordination across health ecosystems (Topic 5: 2.00%). Their perspectives further reflected a focus on governance and policy environments (Topic 2: 16.45%), societal trust and acceptability (Topic 7: 1.17%), and end user needs and relational dynamics (Topic 8: 1.03%).

Regarding opportunities in digital health, Topic 1, accounting for 57.86% of variance, indicated widespread optimism about using digital solutions to enhance health care access, especially via remote care. Topic 2 (7.25%) addressed sustainability and institutional development, and Topic 3 (6.07%) highlighted chronic disease management. Topic 4 (4.04%) explored data-driven, patient-centered innovation, while Topic 5 (3.46%) pointed to implementation gaps at the community level. Subsequent topics included interoperability and workforce readiness (Topic 6: 3.01%), mobile support and training (Topic 7: 1.96%), public infrastructure (Topic 8: 1.80%), epidemic preparedness (Topic 9: 1.54%), and scalable digital records (Topic 10: 1.32%), respectively. In the comparison of male and female responses, both groups emphasized digital health care and remote improvement (male Topic 1: 58.94%; female Topic 1: 66.52%) and sustainability and innovation (male Topic 2: 8.20%; female Topic 2: 9.41%), indicating a shared focus on improving access and strengthening systems. Similarly, training gaps and field limitations were addressed in both analyses (male Topic 7: 2.14%; female Topic 7: 2.11%), as was the management of chronic or communicable diseases, though framed differently: men highlighted mHealth-enabled responses (Topic 3: 7.28%; Topic 5: 3.86%), while women emphasized coordination and surveillance (Topic 3: 5.73%), which was a more diffuse and less prominent theme in men (Topic 8: 1.64%; Topic 10: 1.24%). Differences emerged also in the framing of structural versus relational concerns. Male participants prioritized regional equity and geopolitical targeting (Topics 4 and 9: 4.34% and 1.40%), within a more operational and technology-driven framing of future digital health priorities (Topic 6: 3.34%). Conversely, female respondents focused on patient-centered innovation (Topic 4: 4.47%), interoperability and mHealth gaps (Topic 6: 3.30%; Topic 8: 1.94%; Topic 10: 0.62%), and real-time communication and community linkage (Topic 9: 1.28%), reflecting a more integrative, policy- and user-oriented approach to digital health (Topic 5: 3.69%).

Concerning challenges, Topic 1, accounting for 59.15% of the variance, indicated widespread concern with infrastructural limitations and technological fragmentation. Topic 2 (5.59%) focused on deficits in internet and electricity access, while Topic 3 (3.93%) dealt with implementation bottlenecks in local health systems. Topic 4 (3.71%) addressed access challenges in remote regions, and Topic 5 (3.09%) pointed to fragile community networks and institutional misalignment. Topics 6 and 7 (2.56% and 2.36%, respectively) highlighted digital literacy gaps and rural governance trust issues. Topic 8 (2.18%) was centered on financial constraints, while Topic 9 (1.87%) and Topic 10 (1.63%) underscored politicization and workforce/data infrastructure shortages, respectively. The comparison between male and female LSA topic profiles revealed that both groups identified technological infrastructure and access as the primary concern (male Topic 1: 63.61%; female Topic 1: 45.14%), emphasizing the foundational role of connectivity, electricity, and digital systems. However, male respondents focused more on infrastructural (Topic 2: 5.45%; Topic 8: 1.97%; Topic 10: 1.25%) and professional network gaps (Topic 4: 3.97%; Topic 5: 3.26%), and workforce shortages (Topic 7: 2.36%). In contrast, female respondents emphasized sociopolitical and cultural challenges, including language, policy, and cost barriers (Topic 2: 17.43%; Topic 6: 5.00%), patient access (Topic 3: 8.75%), trust in digital systems (Topic 9: 1.19%), and data protection (Topic 10: 0.89%). Both groups acknowledged rural challenges, education gaps, and politico-economic issues, though women assigned higher variability to these themes (Topic 7: 4.18% vs Topic 7: 2.36%; Topic 5: 6.14% vs Topic 6: 2.80%; Topic 8: 3.02% vs Topic 9: 1.76%; Topic 4: 7.62% vs Topic 3: 4.36%).

In the articulation of future-oriented scenarios, integrated digital care and health technologies (Topic 1: 50.58% explained variance) highlighted a strong consensus on foundational infrastructure and system-wide digital innovation. This was followed by mobile innovation and app-based access (Topic 2: 11.00%) and AI for underserved regions and clinical support (Topic 3: 7.03%), reflecting emphasis on scalable, intelligent solutions for equity and efficiency. Additional themes included electronic health records, mobile health (mHealth), and Internet of Things convergence (Topic 4: 5.26%) and big data potential (Topic 5: 3.43%), both pointing to an evolving interest in interconnected health data ecosystems. Lower-variance topics such as AI-powered decision support (Topic 6: 2.40%), scalable toolkits (Topic 7: 2.13%), and digital medicine (Topic 8: 1.33%) contributed further nuance, underscoring the importance of interoperability, clinical augmentation, and monitoring. For both male and female participants, the dominant theme centered on general digital infrastructure, though with slightly different emphases. Men prioritized general digital health infrastructure (Topic 1: 48.88% variance), emphasizing data and technological systems (Topic 5: 2.99%), while women focused on remote digital health management and records (Topic 1: 72.13%; Topic 7: 1.38%), reflecting a patient-facing orientation grounded in access and care delivery. Male respondents highlighted mobile AI and telemedicine innovation (Topic 2: 13.72%), chronic care and surveillance (Topic 3: 8.98%; Topic 4: 5.10%; Topic 9: 1.38%), and smartphone-based scalable solutions (Topic 6: 2.65%)—a pattern that favors modular, tech-forward tools. In contrast, women showed greater thematic fragmentation across app-based empowerment (Topic 2: 9.17%), AI/chatbot integration with mHealth and Internet of Things (Topic 3: 4.98%; Topic 4: 3.51%; Topic 6: 1.85%), and smartphone planning tools (Topic 8: 0.75%). Notably, chatbot technologies appeared in both profiles (male Topic 7: 2.13%, female Topic 4: 3.51%), although embedded in different semantic contexts—clinical utility for men, and innovation for women. Men also highlighted big data and response systems (Topic 8: 1.56%), while women emphasized real-time data collection (Topic 5: 2.11%).

Further details are reported in Table 1 and Tables S1-S12 in Multimedia Appendix 2.

**Table 1:** Latent semantic analysis–based topic modeling across 4 dimensions of digital health discourse in the Global South: implementation, opportunities, challenges, and foresight. For each of the extracted topics, the percentage of explained variance and corresponding thematic focus are reported.

Topic	Implementation			Opportunities			Challenges			Foresight	
	Explained variance (%)	Thematic focus	Explained variance (%)		Thematic focus	Explained variance (%)		Thematic focus	Explained variance (%)		Thematic focus
1	46.75	Integration, infrastructure, strategic alignment	57.86		Health care access and remote care	59.15		Infrastructural limitations, tech fragmentation	50.58		Integrated digital care and health technologies
2	7.57	Patient management and service delivery	7.25		Sustainability, institutional development	5.59		Internet and electricity access deficits	11.00		Mobile innovation and app-based access
3	4.77	Stakeholder engagement, community outreach	6.07		Chronic disease management	3.93		Local health system bottlenecks	7.03		AI^a^ for underserved regions and clinical support
4	4.21	Governance and regulation	4.04		Data-driven, patient-centered innovation	3.71		Remote region access challenges	5.26		EHR^b^, mHealth, and IoT^c^ convergence
5	4.04	Regulatory clarity and local adaptation	3.46		Implementation gaps at community level	3.09		Community network fragility, institutional misalignment	3.43		Big data potential
6	3.57	Digital literacy and tech access	3.01		Interoperability, workforce readiness	2.56		Digital literacy gaps	2.40		AI-powered clinical decision support
7	2.99	Co-design and participatory processes	1.96		Mobile support and training	2.36		Rural governance trust issues	2.13		Toolkits and scalable tech solutions
8	2.63	Scalability and sustainability	1.80		Public infrastructure	2.18		Financial constraints	1.33		Digital medicine and outcome tracking
9	2.21	Capacity building	1.54		Epidemic preparedness	1.87		Politicization	—^d^		—
10	2.07	Context-aware frameworks	1.32		Scalable digital records	1.63		Workforce/data infrastructure shortages	—		—

^a^AI: artificial intelligence.

^b^EHR: electronic health records.

^c^IoT: Internet of Things.

^d^Not available.

### One Health in the Global South

LSA of responses on One Health opportunities in the Global South revealed a dominant discourse anchored in integrated human, animal, and environmental health systems (Topic 1: 57.20% of the variance). Respondents identified strong opportunities in ecological integration, predictive modeling (Topic 2: 7.27%), digital innovation and AMR surveillance (Topic 3: 6.23%), and AI-driven tools and awareness strategies (Topic 4: 4.79%). Additional themes included prevention and leadership (Topic 5: 3.55%), holistic approaches (Topic 6: 3.10%), community inclusion (Topic 7: 2.29%), vaccine distribution (Topic 8: 1.82%), digital records (Topic 9: 1.60%), and expert capacity building (Topic 10: 1.46%). Female and male respondents shared a strong common emphasis on the ecological foundations of One Health. For both, Topic 1 was dominant—accounting for 73.20% of variance in women and 57.45% in men—and focused on the interconnectedness of human, animal, and environmental health. Beyond this, the thematic priorities diverged significantly. Among women, the discourse was highly concentrated, with secondary themes oriented around strategic implementation and population-level equity. Topic 2 (5.42%) addressed AI and demographic growth, while Topic 3 (4.96%) focused on improving health responses and prevention. Topic 4 (3.93%) indicated attention to sectoral organization. Additional themes included community care and lived experience (Topic 5: 3.73%), treatment applications and clinical uptake (Topic 6: 2.97%), and smart working environments (Topic 7: 2.37%). Less prominent but still present were references to awareness building (Topic 8: 1.23%), health services (Topic 9: 1.14%), and remote training and key local solutions (Topic 10: 0.62%). In contrast, male respondents articulated a more diffuse and operationally driven perspective. Topic 2 (8.27%) emphasized predictive analytics and surveillance technologies, and Topic 3 (7.15%) addressed AMR through a sectoral lens. Topic 4 (5.55%) centered on AI and complex policy coordination. While women focused on communities, men placed greater weight on biodiversity leadership and structured programs (Topic 5: 3.90%), vaccination and disease control strategies (Topic 6: 3.07%), and infectious disease enablement and access (Topic 7: 2.23%). Topic 8 (1.86%) highlighted digital recordkeeping and remote access infrastructure, while Topic 9 (1.60%) captured surveillance, expert knowledge, and vector-borne risks. Topic 10 (1.15%) pointed to health system resources, support, and scale-up potential.

Concerning challenges, Topic 1, accounting for 61.23% of the variance, emphasized environmental degradation, limited resources, and systemic health-environment linkages. Topic 2 (7.43%) highlighted data gaps and weaknesses in surveillance systems, particularly in the context of environmental change. Topic 3 (4.93%) focused on the lack of interdisciplinary coordination and field-level impact. Topic 4 (4.19%) addressed insufficient policy capacity, research infrastructure, and cultural readiness. Topic 5 (3.57%) addressed population-level issues, while Topic 6 (2.76%) pointed to rural marginalization and priority misalignment. Topic 7 (1.97%) underscored digital skill gaps and limited connectivity, and Topic 8 (1.65%) emphasized resource shortages and structural barriers. Topic 9 (1.35%) and Topic 10 (1.29%) captured concerns on meat safety and technological devices. Both female and male respondents strongly converged on structural and environmental limitations as the foremost barrier to implementing One Health in the Global South. In both groups, Topic 1 dominated the discourse—accounting for 61.94% of variance in women and 65.81% in men—and emphasized terms such as health, environment, resource, challenge, and implementation, revealing a shared perception of systemic constraints and infrastructural insufficiency. Beyond this core alignment, the 2 groups diverged notably in the framing and distribution of secondary themes. Among female respondents, Topic 2 (14.68%) focused on population practices, understanding, and adoption, suggesting an emphasis on behavioral, educational, and participatory barriers. Topic 3 (8.84%) addressed funding shortfalls, data gaps, and operational limitations, while Topic 4 (5.32%) pointed to sectoral and institutional weaknesses. Topic 5 (2.36%) dealt with device- and research-based deficiencies. Less prominent themes included poverty (Topic 6: 1.92%), cross-sector coordination (Topic 7: 1.57%), economic emergence and shocks (Topic 8: 1.00%), technological and cultural constraints (Topic 9: 1.00%), and multilevel governance issues (Topic 10: 0.91%). These findings reflect a relatively distributed but coherent concern with systemic functionality, knowledge equity, and integrated governance. In contrast, male respondents articulated a more operationally dispersed set of challenges. Topic 2 (8.80%) dealt with surveillance gaps and environmental degradation, while Topic 3 (5.28%) emphasized capacity building and culturally embedded policy. Topic 4 (3.76%) addressed priority setting and geographic inequities, and Topic 5 (2.98%) brought attention to rural underdevelopment, digital skills, and infrastructure deficits. Topic 6 (2.21%) highlighted low connectivity and staff shortages, and Topic 7 (1.83%) captured community-level implementation difficulties, particularly in relation to meat safety and device availability, while Topic 8 (1.28%) focused on resource and capacity deficits. Topic 9 (1.13%) highlighted disciplinary fragmentation in science and field application, and Topic 10 (1.01%) included conceptual concerns.

Concerning future-related opportunities, Topic 1, explaining 58.78% of the variance, emphasized integrated health approaches linking disease prevention, animal health, and environmental improvement. Topic 2 (5.20%) reflected ambitions for enhanced health care responsiveness through real-time monitoring and information systems. Topic 3 (4.86%) addressed food systems, AMR, and environmental-professional literacy, while Topic 4 (3.80%) pointed to systems development and the implementation of expert-driven frameworks. Additional topics captured emerging or cross-cutting themes. Topic 5 (3.40%) focused on conservation, biodiversity, and domain-specific innovation, and Topic 6 (3.10%) raised concerns about infrastructure, digital records, and electronic health integration. Topic 7 (2.53%) focused on forward-oriented transformative potential of One Health in the Global South. Topic 8 (2.18%) underscored access to care and population equity, while Topic 9 (2.11%) centered on infectious disease awareness and public engagement. Finally, Topic 10 (1.91%) reflected projections around early medical interventions and future modeling scenarios. Male and female respondents both emphasized the ecological nexus of human, animal, and environmental health as a core future opportunity for One Health advancement (male Topic 1: 60.29%; female Topic 1: 68.63%), highlighting a shared foundational understanding across genders. However, differences emerged in the framing and elaboration of these priorities. Women emphasized responsive systems and coordination tools (Topic 2: 20.13%) and showed a stronger orientation toward cultural integration and community empowerment, as seen in topics such as indigenous engagement and awareness promotion (Topic 3: 2.92%), technological advancement and collaboration (Topic 4: 2.77%), and specific innovations like diagnostics (Topic 5: 1.44%), outreach (Topic 6: 0.95%), and data availability (Topic 7: 0.48%). They also included a diffuse but conceptually rich theme on future risks, AMR, and professional preparedness (Topic 8: 0.00%). Male respondents, while similarly prioritizing the health-environment interface (Topic 1: 60.29%), articulated a more technical and operational approach in the remaining topics. These included strengthening sustainable health care systems and food security (Topic 2: 6.03%), implementing expert-led systems (Topic 3: 4.95%), resolving infrastructural and data management issues (Topic 4: 3.93%), and developing digital platforms (Topic 5: 3.33%). Other themes involved community-based research and responsiveness (Topic 6: 3.01%), epidemic readiness and access (Topic 7: 2.69%), early interventions and infectious disease modeling (Topic 8: 2.49%), mobile diagnostics and rapid response (Topic 9: 1.92%), and population-level coordination (Topic 10: 1.61%).

Finally, regarding future-related challenges, Topic 1, explaining 59.65% of the variance, reflected deep-seated anxieties around health system fragility, environmental degradation, resource scarcity, and the growing burden of disease. Topic 2 (7.81%) pointed to digital infrastructure gaps and persistent barriers to health management and equitable access. Topic 3 (5.47%) emphasized food systems, veterinary integration, and the need for cohesive frameworks to support cross-sectoral promotion of One Health. Topic 4 (4.57%) addressed insufficiencies in outbreak detection and relational coordination, while Topic 5 (2.87%) focused on the lack of financial, regulatory, and institutional support for coordinated initiatives. Topic 6 (2.46%) highlighted time-sensitive skill shortages, data deficits, and the limited capacity at the local level. Topic 7 (2.08%) identified environmental opportunities yet to be enabled, suggesting underutilized potential in ecosystem-based approaches. Topic 8 (1.85%) captured cultural and economic inertia that may hinder timely and inclusive responses, and Topic 9 (1.62%) reflected concerns about weak collective action and unrealized potential across populations. Topic 10 (1.41%) pointed to limitations in emergency readiness, agency coordination, and resource mobilization. Both male and female respondents identified systemic and structural limitations as the most significant future challenge for implementing One Health in the Global South. Topic 1 accounted for 78.50% of variance in female responses and 61.65% in men. In both cases, the theme encompassed resource scarcity, environmental degradation, infrastructural weaknesses, and disease burden, indicating a clear consensus on foundational barriers. The female framing included community-based challenges, while the male version emphasized intersectoral and operational breakdowns. Female Topic 2 (8.30%) focused on management, regulatory complexity, cultural barriers, funding deficiencies, and political inertia. This aligns with male Topic 4 (3.42%), which also emphasized coordination, governance, and regulatory support, though framed more technocratically. The female version was more critical and rooted in institutional culture. Female Topic 3 (6.46%) addressed emerging threats, climate-driven disease risk, and resource vulnerabilities, which parallels male Topic 3 (5.77%) on food systems, integration, and outbreak preparedness. Both saw environmental unpredictability and multisectoral fragility as critical future stressors. Female Topic 4 (2.18%) on economic pressure and population-level stress resonated with male Topic 7 (2.13%) and Topic 10 (1.16%), which covered poverty, inequity, local capacity gaps, and leadership voids. The male response provided more layers, incorporating network fragility, population-scale planning, and decentralized leadership, while the female view presented a compact but conceptually broad expression of economic vulnerability. Female Topic 5 (1.39%) emphasized data collection and information deficits, which echoes male Topic 5 (2.80%) and Topic 6 (2.50%) on skills, local-level data systems, and epidemiological responsiveness. The male focus offered more operational detail, while the female version pointed to a diagnostic gap. Female Topic 6 (0.57%) is abstract and thinly developed, but potentially refers to actual lived experience or practical realities—a dimension that, while unelaborated, might align with male Topics 8 (2.01%) and 9 (1.26%) concerning time constraints, remoteness, and pragmatic insufficiencies. Finally, although female Topic 7 (0.00% variance) did not contribute to model variance, it contained rich content including disciplinary fragmentation, outbreak management, and regional opinion dynamics. This latent theme maps well to male Topics 2 (9.17%) and 8 (2.01%), which dealt with Global South–specific digital development, coordination barriers, and interdisciplinary silos.

Further details are reported in Table 2 and Tables S13-S23 in Multimedia Appendix 2.

**Table 2:** Latent semantic analysis–derived topic modeling of stakeholder discourse on One Health in the Global South. Each topic’s explained variance and thematic focus are presented for 4 analytical lenses: opportunities, challenges, future opportunities, and anticipated challenges.

Topic	Opportunities		Challenges		Foresight opportunities		Foresight challenges	
	Explained variance (%)	Thematic focus	Explained variance (%)	Thematic focus	Explained variance (%)	Thematic focus	Explained variance (%)	Thematic focus
1	57.20	Integrated human-animal-environment systems	61.23	Environmental degradation, limited resources, systemic health-environment links	58.78	Integrated disease prevention and environmental improvement	59.65	Health-system fragility, environmental degradation, resource scarcity
2	7.27	Ecological integration, predictive modeling	7.43	Data gaps, surveillance weaknesses	5.2	Real-time monitoring and responsive systems	7.81	Digital infrastructure and access barriers
3	6.23	Digital innovation, AMR^a^ surveillance	4.93	Lack of interdisciplinary coordination	4.86	Food systems, AMR, environmental-professional literacy	5.47	Food systems, veterinary integration, cross-sector support
4	4.79	AI^b^ tools, awareness strategies	4.19	Insufficient policy, research, and cultural capacity	3.8	Systems development, expert frameworks	4.57	Outbreak detection, coordination deficits
5	3.55	Prevention and leadership	3.57	Population-level concerns	3.4	Conservation, biodiversity, innovation	2.87	Financial, regulatory, and institutional voids
6	3.10	Holistic approaches	2.76	Rural marginalization, priority misalignment	3.1	Digital infrastructure, e-records integration	2.46	Skill gaps, data deficits, local capacity
7	2.29	Community inclusion	1.97	Digital skill gaps, limited connectivity	2.53	Transformative One Health potential	2.08	Underutilized ecosystem potential
8	1.82	Vaccine distribution	1.65	Resource shortages, structural barriers	2.18	Access to care, equity	1.85	Cultural/economic inertia
9	1.60	Digital records	1.35	Meat safety concerns	2.11	Infectious disease awareness, engagement	1.62	Weak collective action
10	1.46	Expert capacity building	1.29	Technological device limitations	1.91	Early intervention and predictive modeling	1.41	Emergency readiness, coordination, resource mobilization

^a^AMR: antimicrobial resistance.

^b^AI: artificial intelligence.

### Digital One Health in the Global South

In the LSA of responses to the question on the convergence of digital health and One Health approaches in the Global South, Topic 1, accounting for 75.2% of the total variance, emphasized digitalization, health data infrastructure, and disease surveillance systems, as underpinning the operationalization of the integration of digital health and One Health. Topic 2, explaining 5.4%, captured the role played by global convergence agendas, sectoral alignment, and climate recognition in shaping integrative frameworks across human, animal, and environmental health. Topic 3 (3.9%) pointed to the catalytic effect of the COVID-19 pandemic, which had prompted holistic thinking and increased organizational and financial support for integrated approaches. Topic 4 (3.0%) highlighted how AI and risk management strategies had been mobilized to address systemic constraints and enhance preparedness. Topic 5 (1.8%) reflected the importance of national delivery mechanisms and context-specific implementation, while Topic 6 (1.5%) illustrated the multisectoral impacts that had been particularly salient in the Global South. Topic 7 (1.4%) underscored governance challenges, including institutional fragmentation and reliability gaps, that had historically limited coordinated digital health and One Health responses. Finally, Topics 8 (0.87%), 9 (0.69%), and 10 (0.59%) were centered on policy awareness, stakeholder engagement, and scaling strategies. Topic 1 accounted for the majority of explained variance in both genders: 75.05% in women and 77.31% in men. Both groups emphasized the integration of digital health technologies into disease surveillance within a One Health framework. However, while female responses highlighted the importance of collaborative and community-oriented approaches, male responses were more focused on technological infrastructure and human-disease interaction, illustrating a shared theme with participatory emphasis in women and infrastructural framing in men. Topic 2 was present in both genders (women: 4.77%, men: 5.62%) and concerned management and international drivers of convergence. Female responses were brief and managerial in focus, whereas male responses expanded on sectoral alignment, climate imperatives, and global recognition—pointing to a more externally driven and systemic perspective among male respondents. Topic 3 (women: 4.49%, men: 4.21%) demonstrated alignment around climate change and health system transformation. Women emphasized anticipatory and ecological aspects of convergence, while male responses framed the issue in light of pandemic-triggered reconfigurations and institutional support needs. Both acknowledged the environment-health nexus but with distinct emphases: prospective and global in women, reactive and policy-driven in men. Topic 4 (women: 3.52%, men: 3.24%) centered on institutional governance and risk. Female respondents focused on interministerial communication and vertical coordination, whereas men addressed operational aspects of risk management and the technical requirements of digital health implementation. The shared institutional theme revealed gendered nuances: administrative structure in women versus digital governance in men. Topic 5 (women: 3.26%, men: 1.63%) diverged in scope. Female responses addressed resource allocation and programmatic implementation challenges. In contrast, male discourse shifted toward geopolitical dynamics and multisector imbalances, reflecting broader inequalities in digital readiness across regions. Both pointed to structural enablers and barriers, but from local programmatic (female) and systemic geopolitical (male) vantage points. Topic 6 (women: 2.90%, men: 1.51%) related to delivery and performance. Women focused on access and service efficacy, while men underlined fragmentation and interministerial unreliability. Both acknowledged weaknesses in execution, with women emphasizing outcomes and men highlighting administrative dysfunction. Topic 7 was marginal among women (1.59%) and absent in the male profile. The theme suggested a latent concern with public engagement and legitimacy, though it remained underdeveloped in both groups. Topic 8 (women: 1.46%, men: 0.64%) touched on data access and informational equity. Both groups acknowledged the enabling role of digital health in democratizing health information, although the female response focused more explicitly on availability and transparency. Topic 9 (women: 1.45%, men: 0.59%) was conceptually minimal. While women pointed to the importance of key factors without elaboration, male responses offered no interpretable content. This suggests a shared but weakly articulated recognition of critical convergence enablers. Topic 10 was notably richer in the female discourse (0.78% vs 0.51%). Female responses addressed themes such as health inequity, underserved populations, antimicrobial stewardship, and system efficiency. Male responses, in contrast, referred to national context or jurisdiction.

Further details are reported in Table 3 and Tables S24-S26 in Multimedia Appendix 2.

**Table 3:** Latent semantic analysis of stakeholder responses on the convergence of digital health and One Health in the Global South.

Topic	Explained variance (%)	Thematic focus
1	75.2	Digitalization, health data infrastructure, disease surveillance
2	5.4	Global convergence agendas, sectoral alignment, climate recognition
3	3.9	Catalytic effect of COVID-19 on holistic and integrative thinking
4	3.0	AI^a^ and risk management strategies for systemic preparedness
5	1.8	National delivery mechanisms and context-specific implementation
6	1.5	Multisectoral impacts in the Global South
7	1.4	Governance challenges and institutional fragmentation
8	0.87	Policy awareness and advocacy
9	0.69	Stakeholder engagement
10	0.59	Scaling strategies

^a^AI: artificial intelligence.

## Discussion

### Principal Findings

The foresight-oriented analysis of expert narratives revealed distinct thematic architectures underlying the discourses on digital health, One Health, and their convergence in the Global South. Importantly, these architectures should be interpreted not in isolation, but in relation to the existing international literature, which has been overwhelmingly shaped by Global North perspectives.

In the domain of digital health, structural drivers were consistently anchored in the infrastructural and systemic integration of digital technologies into health systems. These included the entrenchment of digital data ecosystems, the scaling of mHealth interventions, and the embedding of digital platforms into clinical workflows and public health delivery. Such drivers reflect a maturing landscape where digital technologies are no longer peripheral but fundamental to the functioning and reform of health governance in many Global South contexts. This emphasis partially mirrors findings from the Global North, where digital health maturity is typically framed around interoperability, large-scale electronic health records, and AI-enabled clinical decision support embedded within stable institutional environments. However, unlike Global North settings—where digital infrastructure is often treated as a given—the Global South discourse consistently foregrounds infrastructural fragility, uneven access to electricity and connectivity, and dependence on external funding as persistent structural constraints.

Emerging issues in digital health centered on the transformation of care models through AI, the proliferation of mobile diagnostics, and evolving concerns around data sovereignty and ethical oversight. These topics reflect a transition in focus from basic digital inclusion toward strategic, anticipatory integration of advanced technologies. While similar themes appear in Global North studies—particularly around AI adoption and data governance—these findings suggest a qualitatively different framing in the Global South, where AI is often viewed as an aspirational or compensatory tool intended to offset workforce shortages and system limitations, rather than as an optimization layer built upon already robust health systems.

Particularly noteworthy was the shift from discussions of access and deployment to themes of agency, responsiveness, and the societal implications of algorithmic decision-making. Gendered nuances were evident, with female respondents frequently framing these issues through the lenses of equity, community participation, and trust, while male respondents emphasized operational scalability and innovation efficiency.

Within the One Health discourse, structural drivers coalesced around intersectoral coordination, disease surveillance at the human-animal-environment interface, and the growing imperative to institutionalize One Health frameworks. These reflect a stable recognition of ecological entanglement and the necessity of integrative governance structures to mitigate transboundary health threats. The discourse was marked by convergent priorities such as early warning systems, environmental health data infrastructures, and capacity-building in veterinary and environmental public health. Across genders, these priorities were consistently articulated, although women more frequently situated them within community resilience and social vulnerability contexts. This aligns with Global North One Health literature, which similarly prioritizes multisectoral surveillance, zoonotic preparedness, and early warning systems. However, in contrast to Global North contexts—where One Health is often operationalized through formalized interagency mechanisms—the Global South discourse reveals that coordination remains largely aspirational, constrained by fragmented mandates, limited regulatory authority, and underresourced environmental and veterinary sectors.

Emerging issues in One Health focused on anticipatory risk management, the operationalization of multisectoral collaborations, and the rising salience of food system sustainability and climate resilience. These signal a discursive broadening of One Health from zoonotic disease control toward more systemic, planetary health considerations. Notably, whereas Global North literature increasingly frames these challenges through the lens of technological integration and policy alignment, Global South experts more frequently emphasized structural underinvestment, competing development priorities, and the vulnerability of rural and marginalized populations to ecological shocks.

In several instances, female participants extended the discourse into areas of inclusive governance, highlighting the importance of participatory institutions and local knowledge systems in shaping resilient One Health infrastructures. These contributions suggest a shift toward democratized models of One Health governance that move beyond technical coordination toward transformative system change. Weak signals in One Health included fragmented reflections on indigenous knowledge integration, AMR governance at subnational levels, and structural underinvestment in ecological public health. Though sparse in expression, these signals may indicate latent areas of strategic neglect that could become critical under future scenarios marked by climate instability or political realignment. Such weak signals are largely absent or marginal in the Global North literature, where One Health discussions tend to privilege formal scientific expertise and national-level governance, thereby underscoring the added value of a foresight-oriented approach capable of surfacing peripheral yet potentially transformative concerns. The gendered content of these weak signals was particularly rich, with women surfacing relational, trust-based framings and men occasionally noting the absence of jurisdictional clarity and institutional mandates.

The discourse on the convergence of digital health and One Health was anchored by structural drivers that framed integration as both inevitable and strategic. Participants consistently viewed digital tools as essential enablers of One Health implementation, particularly in data sharing, interagency coordination, and real-time surveillance. This convergence was portrayed not as a mere overlay of technologies but as a paradigmatic shift toward more intelligent, interconnected, and adaptive health ecosystems. Emerging issues in the convergence discourse included the governance of cross-sectoral data flows, the development of digital infrastructures tailored to ecological surveillance, and the political economy of technology access in Global South contexts. These themes reflected a growing awareness of the nonneutrality of digital infrastructures and the need to align technological solutions with values of justice, inclusion, and environmental stewardship. Gendered patterns again surfaced, with female experts more likely to emphasize coproduction, relational accountability, and the risks of epistemic exclusion. This mirrors Global North narratives that conceptualize Digital One Health primarily as a technical convergence problem. However, the Global South discourse identified here reframes convergence as a deeply political and sociotechnical process, shaped by digital sovereignty, power asymmetries, and unequal access to technological resources.

Weak signals in the convergence space included underexplored themes such as feminist digital ethics, algorithmic bias in zoonotic prediction models, and the political implications of digital sovereignty in environmental health governance. Though peripheral in current discourse, these signals point to potentially disruptive tensions that could reshape future directions in Digital One Health if left unaddressed. Their emergence in this study highlights how foresight methodologies, particularly when combined with gender-sensitive analysis, can reveal dimensions of convergence that are systematically overlooked in implementation-focused research.

Altogether, the analysis surfaced a dynamic and increasingly reflexive ecosystem of discourse, in which well-established priorities coexist with early indicators of transformative change. By explicitly contrasting Global South expert imaginaries with the dominant Global North literature, this study demonstrates that Digital One Health convergence is not a universally linear or technologically deterministic process. Rather, it is contextually negotiated, unevenly paced, and deeply shaped by structural inequities, governance capacities, and gendered epistemologies. By disaggregating structural drivers, emerging issues, and weak signals across gendered and thematic lines, the study contributes a nuanced, equity-informed foresight mapping of how health futures are being envisioned in the Global South.

Building upon the LSA-based foresight mapping, a comparative reflection with recent qualitative literature [14-17] on digital health and One Health in the Global South reveals substantial areas of alignment as well as conceptual expansion. While prior studies have independently captured infrastructural, systemic, and organizational dimensions, this LSA analysis contributes temporal depth and a gendered, anticipatory framing that enriches and challenges existing scholarship.

### Strengths and Limitations

This study leverages a robust methodological approach by integrating LSA-based topic modeling with foresight-oriented frameworks to explore the convergence of digital health and One Health in the Global South. The analytical design, which mapped present and future-oriented discourses across 4 strategic dimensions—implementation, opportunities, challenges, and prospective trajectories—enabled a layered classification of structural drivers, emerging issues, and weak signals. The incorporation of a Delphi-informed purposive sampling strategy ensured the inclusion of a diverse array of expert voices across geographical, disciplinary, and institutional boundaries. This methodological rigor lends robustness and ecological validity to the findings, especially in capturing the pluralistic and multisectoral character of the digital health and One Health convergence discourse in resource-constrained settings.

Despite these strengths, the study is not without limitations. First, while purposive sampling captured disciplinary diversity, the geographic composition of the expert panel was heavily skewed toward Africa, with 58% (26/45) of participants based in African countries. In contrast, representation from Latin America and the Caribbean and the Middle East and North Africa was minimal, comprising only 7% (3/45) and 4% (2/45) of the sample, respectively. Due to this imbalance, it was not possible to perform a reliable LSA stratified by geographic provenance. As such, important regional nuances—especially from underrepresented areas—may not be adequately captured. Second, the reliance on English-language responses potentially excluded non-Anglophone voices and introduced language-related semantic constraints. Third, while LSA effectively reduces linguistic dimensionality and enables the identification of latent thematic structures, it may obscure context-specific narrative nuance and is sensitive to preprocessing decisions such as tokenization, term selection, and sparsity thresholds. As a result, the derived topics reflect dominant semantic patterns rather than the full richness or contextual specificity of individual expert narratives, and should be interpreted as heuristic representations rather than definitive constructs. Finally, although the study incorporated a gender lens, the male-to-female ratio was 2.8:1, limiting the interpretive depth of gender-divergent discourse, particularly for weak signals.

A further limitation relates to the reliance on open-ended survey questions. While this approach was intentionally adopted to support exploratory, foresight-oriented inquiry, it inevitably introduces interpretive variability, as respondents may construe and frame the same prompt in different ways. In this study, this variability was reflected in systematic differences between male and female respondents, not only in the thematic priorities they emphasized but also in the semantic framing and level of abstraction of their responses. Such gender-related divergences should not be interpreted as inconsistencies or measurement error, but rather as an inherent feature of qualitative, discursive data elicited through open-ended instruments. Nonetheless, this interpretive flexibility limits direct comparability across respondent groups and constrains inferential interpretations of gender differences. While the use of LSA helped to identify shared latent structures across heterogeneous narratives, it cannot fully eliminate variability arising from differential interpretation of survey prompts. Closely related to this issue, an additional limitation concerns the survey instrument itself. The questionnaire was developed ad hoc for this study and was not based on a previously validated instrument. This approach was necessary given the exploratory and foresight-oriented nature of the research, as well as the absence of established instruments designed to capture expert perspectives on the convergence of digital health and One Health, particularly in Global South contexts. However, the use of an ad hoc instrument limits direct comparability with other studies and may affect measurement consistency. The multiround Delphi design, purposive expert sampling, and the application of LSA partially mitigated this limitation by emphasizing convergence of meaning across narratives rather than item-level psychometric properties. In addition, the predominance of midcareer participants—while purposeful for capturing forward-looking and implementation-oriented perspectives—may have limited the extent to which long-term institutional memory and historical continuity were represented. Future research could address these limitations through more focused and structured study designs. Follow-up studies may combine open-ended foresight prompts with semistructured or closed-ended items anchored to predefined conceptual domains such as governance, infrastructure, ethics, and equity. The use of scenario-based questions, vignettes, or Likert-scaled assessments of clearly defined drivers and barriers could enhance cross-group comparability while preserving interpretive depth. Mixed methods approaches integrating qualitative elicitation with quantitative validation, alongside more balanced gender representation, would further strengthen the robustness, comparability, and interpretability of future analyses.

### Conclusions

This study provides a foresight-informed, gender-sensitive mapping of the convergence between digital health and One Health discourses in the Global South. Structural drivers centered on the systemic integration of digital infrastructure, intersectoral governance, and ecological surveillance, while emerging issues pointed toward anticipatory innovation, AI deployment, and food system resilience. Weak signals—particularly those surfaced by female respondents—highlighted underexplored dimensions such as indigenous knowledge, feminist digital ethics, and community-based relational dynamics. By disaggregating expert discourse through LSA and embedding it within a futures thinking framework, the study surfaces both prevailing and nascent imaginaries shaping global health trajectories. These findings underscore the importance of inclusive, equity-oriented approaches in shaping Digital One Health agendas, and call for stronger attention to gendered epistemologies, participatory governance, and digital sovereignty in future research and implementation efforts.

## Appendix

Multimedia Appendix 1 Survey items.

Multimedia Appendix 2 Additional tables.

## Abbreviations

AI: artificial intelligence
AMR: antimicrobial resistance
LSA: latent semantic analysis
mHealth: mobile health

## References

[1] Barrett B, Charles JW, Temte JL (2015). Climate change, human health, and epidemiological transition. Prev Med.

[2] Hua K, Pan Y, Fang J, Wu H, Hua Y (2024). Integrating social, climate and environmental changes to confront accelerating global aging. BMC Public Health.

[3] Boutayeb A (2010). The burden of communicable and non-communicable diseases in developing countries. Handbook of Disease Burdens and Quality of Life Measures.

[4] Acolin J, Fishman P (2023). Beyond the biomedical, towards the agentic: a paradigm shift for population health science. Soc Sci Med.

[5] Horefti E (2023). The importance of the One Health concept in combating zoonoses. Pathogens.

[6] Danasekaran R (2024). One Health: a holistic approach to tackling global health issues. Indian J Community Med.

[7] Abernethy A, Adams L, Barrett M, Bechtel C, Brennan P, Butte A, Faulkner J, Fontaine E, Friedhoff S, Halamka J, Howell M, Johnson K, Long P, McGraw D, Miller R, Lee P, Perlin J, Rucker D, Sandy L, Savage L, Stump L, Tang P, Topol E, Tuckson R, Valdes K (2022). The promise of digital health: then, now, and the future. NAM Perspect.

[8] Scott P, Adedeji T, Nakkas H, Andrikopoulou E (2023). One Health in a digital world: technology, data, information and knowledge. Yearb Med Inform.

[9] Benis A, Tamburis O, Chronaki C, Moen A (2021). One Digital Health: a unified framework for future health ecosystems. J Med Internet Res.

[10] Ragnedda M, Gladkova A, Ragnedda M, Gladkova A (2020). Understanding digital inequalities in the Gobal South. Digital Inequalities in the Global South. Global Transformations in Media and Communication Research - A Palgrave and IAMCR Series.

[11] Rezaeian M, Montazeri H, Loonen RCGM (2017). Science foresight using life-cycle analysis, text mining and clustering: a case study on natural ventilation. Technol Forecast Soc Change.

[12] Kayser V, Blind K (2017). Extending the knowledge base of foresight: the contribution of text mining. Technol Forecast Soc Change.

[13] OECD (2025). Strategic Foresight Toolkit for Resilient Public Policy: A Comprehensive Foresight Methodology to Support Sustainable and Future-Ready Public Policy.

[14] O'Brien N, Li E, Chaibva CN, Gomez Bravo R, Kovacevic L, Kwame Ayisi-Boateng N, Lounsbury O, Nwabufo NFF, Senkyire EK, Serafini A, Surafel Abay E, van de Vijver S, Wanjala M, Wangari M, Moosa S, Neves AL (2023). Strengths, weaknesses, opportunities, and threats analysis of the use of digital health technologies in primary health care in the sub-Saharan African region: qualitative study. J Med Internet Res.

[15] Steenkamp I, Peltonen LM, Chipps J (2025). Digital health readiness - insights from healthcare leaders in operational management: a cross-sectional survey. BMC Health Serv Res.

[16] Zharima C, Griffiths F, Goudge J (2023). Exploring the barriers and facilitators to implementing electronic health records in a middle-income country: a qualitative study from South Africa. Front Digit Health.

[17] Brown HL, Pursley IG, Horton DL, La Ragione RM (2024). One Health: a structured review and commentary on trends and themes. One Health Outlook.

