# Supplementary material for: Framing the Convergence of One Health and Digital Health in the Global South With a Gender-Sensitive Foresight Perspective: Delphi Study Using Latent Semantic Analysis
Source: J Med Internet Res. 2026 Feb 18;28:e78702. doi: 10.2196/78702 (PMC12961381; doi:10.2196/78702)
Supplement: Multimedia Appendix 1 [file jmir_v28i1e78702_app1.docx]

**Appendix 1. Delphi Survey Instrument on the Convergence of Digital Health and One Health in the Global South.**

Section 1. Sociodemographic Information

1. Age (years)
2. Gender
3. Country in which you were born
4. Country or countries in which you have studied
5. Current country you are living in
6. Current professional position
7. Area(s) of expertise in digital health and/or One Health
8. Years of expertise in digital health and/or One Health
9. How interdisciplinary would you define your current working environment (in terms of collaborations among healthcare professionals, veterinarians, environmental scientists, and technologists)?

Section 2. Digital Health in the Global South

1. In your opinion, what are the most significant opportunities in the field of digital health in your country and, more broadly, in the Global South today?
2. In your opinion, what are the most significant challenges in the field of digital health in your country and, more broadly, in the Global South today?
3. Which digital health technologies do you believe will be most impactful in healthcare delivery and access in your country and the Global South in the coming decade(s)?
4. What digital health initiatives or projects have you been involved in or are aware of in your region? Please describe them briefly.

Section 3. One Health in the Global South

1. In your opinion, what are the most significant opportunities in the field of One Health in your country and, more broadly, in the Global South today?
2. In your opinion, what are the most significant challenges in the field of One Health in your country and, more broadly, in the Global South today?
3. In your opinion, what are the most significant opportunities in the field of One Health in your country and, more broadly, in the Global South in the coming decades?
4. In your opinion, what are the most significant challenges in the field of One Health in your country and, more broadly, in the Global South in the coming decades?
5. What One Health initiatives or projects have you been involved in or are aware of in your region? Please describe them briefly.

Section 4. Convergence and Strategic Foresight

1. What key factors do you believe are driving the convergence of digital health and One Health approaches in addressing health challenges in your country and, more broadly, the Global South?
2. Please provide any additional comments or insights that you believe are important for shaping the future of digital health and One Health in your country and, more broadly, in the Global South.
3. Since you are asked to take part in following rounds, please generate a unique code that we will use for matching, while preserving the anonymous status of this survey. Compose your code with the initial of your favourite colour, the first three letters of your birth city, and the last two digits of your phone number.
