# Supplementary material for: Framing the Convergence of One Health and Digital Health in the Global South With a Gender-Sensitive Foresight Perspective: Delphi Study Using Latent Semantic Analysis
Source: J Med Internet Res. 2026 Feb 18;28:e78702. doi: 10.2196/78702 (PMC12961381; doi:10.2196/78702)
Supplement: Multimedia Appendix 2 [file jmir_v28i1e78702_app2.docx]

**Table S1. Latent Semantic Analysis-based topic modelling across the implementation dimension of digital health discourse in the Global South. For each of the extracted topics, the percentage of explained variance, number of terms, and terms are reported.**

| **Topic** | **Eigenvalue** | **Explained variance (%)** | **Number of Terms** | **Terms** |
| --- | --- | --- | --- | --- |
| Topic 1 | 25.775 | 46.752 | 67 | digit, health, solut, healthcar, implement |
| Topic 2 | 10.368 | 7.565 | 20 | data, patient, manag, servic, care |
| Topic 3 | 8.236 | 4.774 | 11 | engag, educ, privat, term, hing |
| Topic 4 | 7.735 | 4.211 | 4 | ministri, regul, maker, project |
| Topic 5 | 7.578 | 4.041 | 9 | regulatori, reliabl, area, easi, depend |
| Topic 6 | 7.118 | 3.566 | 8 | literaci, devic, case, secur, qualiti |
| Topic 7 | 6.527 | 2.998 | 4 | design, continu, player, particip |
| Topic 8 | 6.107 | 2.625 | 10 | scalabl, sustain, strong, intern, leverag |
| Topic 9 | 5.597 | 2.205 | 5 | resourc, human, awar, capabl, expert |
| Topic 10 | 5.427 | 2.072 | 10 | work, countri, framework, problem, societi |

**Table S2. Latent Semantic Analysis-based topic modelling across the opportunity dimension of digital health discourse in the Global South. For each of the extracted topics, the percentage of explained variance, number of terms, and terms are reported.**

| **Topic** | **Eigenvalue** | **Explained variance (%)** | **Number of Terms** | **Terms** |
| --- | --- | --- | --- | --- |
| Topic 1 | 28.874 | 57.857 | 61 | health, digit, healthcar, improv, remot |
| Topic 2 | 10.217 | 7.245 | 9 | develop, sustain, achiev, foster, resourc |
| Topic 3 | 9.352 | 6.069 | 11 | diseas, communic, burden, app, diabet |
| Topic 4 | 7.632 | 4.042 | 7 | patient, expand, util, analysi, advanc |
| Topic 5 | 7.065 | 3.464 | 12 | communiti, gap, countri, bridg, practic |
| Topic 6 | 6.591 | 3.014 | 3 | address, interoper, workforc |
| Topic 7 | 5.311 | 1.958 | 6 | train, qualiti, insuffici, field, phone |
| Topic 8 | 5.095 | 1.802 | 12 | univers, capac, applic, public, build |
| Topic 9 | 4.706 | 1.537 | 3 | epidem, growth, knowledg |
| Topic 10 | 4.368 | 1.324 | 7 | electron, record, intervent, reach, time |

**Table S3. Latent Semantic Analysis-based topic modelling across the challenge dimension of digital health discourse in the Global South. For each of the extracted topics, the percentage of explained variance, number of terms, and terms are reported.**

| **Topic** | **Eigenvalue** | **Explained variance (%)** | **Number of Terms** | **Terms** |
| --- | --- | --- | --- | --- |
| Topic 1 | 25.922 | 59.148 | 65 | digit, health, healthcar, data, technolog |
| Topic 2 | 7.968 | 5.589 | 10 | internet, electr, infrastructur, polici, low |
| Topic 3 | 6.683 | 3.932 | 6 | implement, capac, local, pose, countri |
| Topic 4 | 6.487 | 3.705 | 5 | care, network, remot, signific, develop |
| Topic 5 | 5.924 | 3.089 | 5 | avail, collabor, communiti, resist, structur |
| Topic 6 | 5.397 | 2.564 | 3 | mobil, educ, expand |
| Topic 7 | 5.176 | 2.358 | 11 | rural, model, environ, trust, govern |
| Topic 8 | 4.978 | 2.181 | 5 | connect, poor, financi, time, spread |
| Topic 9 | 4.609 | 1.870 | 2 | widespread, polit |
| Topic 10 | 4.306 | 1.632 | 4 | personnel, nation, comput, dataset |

**Table S4. Latent Semantic Analysis-based topic modelling across the foresight dimension of digital health discourse in the Global South. For each of the extracted topics, the percentage of explained variance, number of terms, and terms are reported.**

| **Topic** | **Eigenvalue** | **Explained variance (%)** | **Number of Terms** | **Label** | **Terms** |
| --- | --- | --- | --- | --- | --- |
| Topic 1 | 20.976 | 50.576 | 62 | Integrated digital care and health technologies | health, patient, healthcar, technolog, medic |
| Topic 2 | 9.782 | 10.999 | 4 | Mobile innovation and app-based access | innov, signific, app, make |
| Topic 3 | 7.821 | 7.030 | 18 | AI for underserved regions and clinical support | improv, ai, enabl, area, underserv |
| Topic 4 | 6.762 | 5.256 | 5 | EHR, mHealth, and IoT convergence | resourc, ehr, mhealth, scienc, iot |
| Topic 5 | 5.460 | 3.427 | 1 | Big data potential | big |
| Topic 6 | 4.568 | 2.398 | 8 | AI-powered clinical decision support | base, databas, doctor, assist, larg |
| Topic 7 | 4.302 | 2.127 | 3 | Toolkits and scalable tech solutions | tool, power, solut |
| Topic 8 | 3.397 | 1.327 | 3 | Digital medicine and outcome tracking | medicin, respons, collect |

**Table S5. Latent Semantic Analysis-based topic modelling across the implementation dimension of digital health discourse in the Global South in female respondents. For each of the extracted topics, the percentage of explained variance, number of terms, and terms are reported.**

| **Topic** | **Eigenvalue** | **Explained variance (%)** | **Number of Terms** | **Terms** |
| --- | --- | --- | --- | --- |
| 1 | 12.328 | 52.95 | 52 | health, digit, polici, infrastructur, communiti |
| 2 | 6.871 | 16.45 | 9 | ministri, medic, maker, record, electron |
| 3 | 5.145 | 9.22 | 15 | train, human, capabl, public, crucial |
| 4 | 3.276 | 3.74 | 5 | support, research, financi, depend, project |
| 5 | 2.393 | 2.00 | 3 | network, player, make |
| 6 | 2.118 | 1.56 | 2 | strategi, align |
| 7 | 1.830 | 1.17 | 2 | societi, trust |
| 8 | 1.715 | 1.03 | 3 | friend, user, resourc |
| 9 | 1.547 | 0.83 | 57 | patient, personnel, clear, ecosystem, diseas |

**Table S6. Latent Semantic Analysis-based topic modelling across the implementation dimension of digital health discourse in the Global South in male respondents. For each of the extracted topics, the percentage of explained variance, number of terms, and terms are reported.**

| **Topic** | **Eigenvalue** | **Explained variance (%)** | **Number of Terms** | **Terms** |
| --- | --- | --- | --- | --- |
| 1 | 23.855 | 50.18 | 60 | digit, health, solut, healthcar, implement |
| 2 | 9.834 | 8.53 | 21 | patient, data, manag, servic, care |
| 3 | 7.606 | 5.10 | 13 | afford, user, cost, regulatori, devic |
| 4 | 7.166 | 4.53 | 2 | literaci, case |
| 5 | 6.647 | 3.90 | 4 | continu, sector, depend, worker |
| 6 | 6.183 | 3.37 | 5 | support, strong, secur, awar, project |
| 7 | 5.510 | 2.68 | 12 | work, countri, resourc, leverag, organ |
| 8 | 5.165 | 2.35 | 9 | internet, communiti, easi, benefit, personnel |
| 9 | 5.018 | 2.22 | 11 | adopt, framework, qualiti, network, broader |
| 10 | 4.893 | 2.11 | 11 | promot, privat, robust, ministri, financi |

**Table S7. Latent Semantic Analysis-based topic modelling across the opportunity dimension of digital health discourse in the Global South in female respondents. For each of the extracted topics, the percentage of explained variance, number of terms, and terms are reported.**

| **Topic** | **Eigenvalue** | **Explained variance (%)** | **Number of Terms** | **Terms** |
| --- | --- | --- | --- | --- |
| 1 | 13.841 | 66.52 | 51 | health, healthcar, digit, data, improv |
| 2 | 5.204 | 9.41 | 7 | manag, diseas, app, electron, record |
| 3 | 4.064 | 5.73 | 4 | analysi, surveil, coordin, chronic |
| 4 | 3.586 | 4.47 | 5 | patient, connect, nigeria, rapid, profession |
| 5 | 3.261 | 3.69 | 1 | strategi |
| 6 | 3.084 | 3.30 | 5 | care, growth, medic, establish, tool |
| 7 | 2.466 | 2.11 | 3 | knowledg, bring, countri |
| 8 | 2.365 | 1.94 | 2 | drive, effici |
| 9 | 1.923 | 1.28 | 4 | transfer, real, time, communiti |
| 10 | 1.339 | 0.62 | 49 | reach, addit, interoper, mhealth, insuffici |

**Table S8. Latent Semantic Analysis-based topic modelling across the opportunity dimension of digital health discourse in the Global South in male respondents. For each of the extracted topics, the percentage of explained variance, number of terms, and terms are reported.**

| **Topic** | **Eigenvalue** | **Explained variance (%)** | **Number of Terms** | **Terms** |
| --- | --- | --- | --- | --- |
| 1 | 26.068 | 58.94 | 55 | health, digit, healthcar, improv, remot |
| 2 | 9.725 | 8.20 | 16 | develop, sustain, resourc, innov, electron |
| 3 | 9.158 | 7.28 | 8 | diseas, communic, burden, app, adopt |
| 4 | 7.070 | 4.34 | 7 | gap, south, global, bridg, leverag |
| 5 | 6.670 | 3.86 | 13 | communiti, patient, address, enhanc, mhealth |
| 6 | 6.209 | 3.34 | 4 | advanc, expand, util, streamlin |
| 7 | 4.968 | 2.14 | 4 | train, qualiti, insuffici, phone |
| 8 | 4.345 | 1.64 | 2 | epidem, growth |
| 9 | 4.013 | 1.40 | 12 | field, africa, intervent, connect, nigeria |
| 10 | 3.778 | 1.24 | 10 | diabet, monitor, build, infecti, surveil |

**Table S9. Latent Semantic Analysis-based topic modelling across the challenge dimension of digital health discourse in the Global South in female respondents. For each of the extracted topics, the percentage of explained variance, number of terms, and terms are reported.**

| **Topic** | **Eigenvalue** | **Explained variance (%)** | **Number of Terms** | **Terms** |
| --- | --- | --- | --- | --- |
| 1 | 8.310 | 45.14 | 36 | digit, health, internet, electr, healthcar |
| 2 | 5.164 | 17.43 | 10 | languag, polici, cost, widespread, fear |
| 3 | 3.660 | 8.75 | 7 | patient, access, structur, weak, build |
| 4 | 3.414 | 7.62 | 5 | lack, insuffici, resist, fund, provid |
| 5 | 3.065 | 6.14 | 7 | educ, spread, expand, countri, comput |
| 6 | 2.765 | 5.00 | 3 | network, avail, cultur |
| 7 | 2.528 | 4.18 | 2 | rural, popul |
| 8 | 2.150 | 3.02 | 4 | polit, econom, issu, challeng |
| 9 | 1.348 | 1.19 | 1 | trust |
| 10 | 1.164 | 0.89 | 41 | clinic, dataset, outcom, protect, promot |

**Table S10. Latent Semantic Analysis-based topic modelling across the challenge dimension of digital health discourse in the Global South in male respondents. For each of the extracted topics, the percentage of explained variance, number of terms, and terms are reported.**

| **Topic** | **Eigenvalue** | **Explained variance (%)** | **Number of Terms** | **Terms** |
| --- | --- | --- | --- | --- |
| 1 | 25.005 | 63.61 | 58 | digit, health, healthcar, data, technolog |
| 2 | 7.316 | 5.45 | 9 | internet, infrastructur, electr, area, local |
| 3 | 6.543 | 4.36 | 6 | implement, pose, countri, resist, clinic |
| 4 | 6.249 | 3.97 | 8 | profession, polici, network, remot, mobil |
| 5 | 5.658 | 3.26 | 3 | avail, collabor, communiti |
| 6 | 5.247 | 2.80 | 6 | low, care, educ, expand, insuffici |
| 7 | 4.814 | 2.36 | 8 | rural, model, make, trust, shortag |
| 8 | 4.395 | 1.97 | 8 | connect, poor, weak, spread, time |
| 9 | 4.164 | 1.76 | 4 | general, personnel, financi, widespread |
| 10 | 3.510 | 1.25 | 6 | resourc, build, govern, nation, promot |

**Table S11. Latent Semantic Analysis-based topic modelling across the foresight dimension of digital health discourse in the Global South in female respondents. For each of the extracted topics, the percentage of explained variance, number of terms, and terms are reported.**

| **Topic** | **Eigenvalue** | **Explained Variance (%)** | **Number of Terms** | **Terms** |
| --- | --- | --- | --- | --- |
| 1 | 12.395 | 72.130 | 58 | health, medic, remot, manag, record |
| 2 | 4.420 | 9.172 | 3 | app, signific, power |
| 3 | 3.257 | 4.982 | 4 | medicin, platform, doctor, make |
| 4 | 2.735 | 3.513 | 5 | ai, chatbot, innov, mhealth, iot |
| 5 | 2.120 | 2.110 | 2 | instant, collect |
| 6 | 1.985 | 1.851 | 1 | telehealth |
| 7 | 1.711 | 1.375 | 2 | analysi, larg |
| 8 | 1.263 | 0.749 | 29 | solut, base, smartphon, plan, scienc |

**Table S12. Latent Semantic Analysis-based topic modelling across the foresight dimension of digital health discourse in the Global South in male respondents. For each of the extracted topics, the percentage of explained variance, number of terms, and terms are reported.**

| **Topic** | **Eigenvalue** | **Explained Variance (%)** | **Number of Terms** | **Terms** |
| --- | --- | --- | --- | --- |
| 1 | 17.920 | 48.877 | 50 | health, patient, healthcar, technolog, data |
| 2 | 9.496 | 13.724 | 14 | mobil, ai, telemedicin, diseas, innov |
| 3 | 7.680 | 8.976 | 17 | enabl, chronic, record, prevent, surveil |
| 4 | 5.786 | 5.095 | 1 | detect |
| 5 | 4.432 | 2.990 | 9 | databas, base, effici, promot, work |
| 6 | 4.176 | 2.654 | 4 | smartphon, expand, solut, power |
| 7 | 3.737 | 2.126 | 5 | tool, applic, chatbot, resourc, medicin |
| 8 | 3.205 | 1.564 | 3 | big, respons, collect |
| 9 | 3.011 | 1.380 | 1 | assist |

**Table S13. Latent Semantic Analysis-based topic modelling across the opportunity dimension of one health discourse in the Global South. For each of the extracted topics, the percentage of explained variance, number of terms, and terms are reported.**

| **Topic** | **Eigenvalue** | **Explained Variance (%)** | **Number of Terms** | **Terms** |
| --- | --- | --- | --- | --- |
| 1 | 25.084 | 57.20 | 63 | health, disease, human, environment, anim |
| 2 | 8.940 | 7.27 | 11 | predict, monitor, model, manag, smart |
| 3 | 8.279 | 6.23 | 11 | field, digit, antimicrobi, resist, sector |
| 4 | 7.257 | 4.79 | 5 | research, ai, awar, servic, tool |
| 5 | 6.249 | 3.55 | 8 | prevent, control, lead, invest, engag |
| 6 | 5.838 | 3.10 | 2 | holist, high |
| 7 | 5.016 | 2.29 | 5 | peopl, communiti, access, live, univers |
| 8 | 4.474 | 1.82 | 4 | anthrax, vaccin, support, distribut |
| 9 | 4.196 | 1.60 | 10 | inform, record, electron, care, remot |
| 10 | 4.006 | 1.46 | 3 | expert, grow, increas |

**Table S14. Latent Semantic Analysis-based topic modelling across the challenge dimension of one health discourse in the Global South. For each of the extracted topics, the percentage of explained variance, number of terms, and terms are reported.**

| **Topic** | **Eigenvalue** | **Explained Variance (%)** | **Number of Terms** | **Terms** |
| --- | --- | --- | --- | --- |
| 1 | 25.452 | 61.229 | 55 | health, environment, limit, challeng, resourc |
| 2 | 8.866 | 7.429 | 21 | surveil, data, includ, chang, degrad |
| 3 | 7.223 | 4.931 | 8 | address, communiti, interdisciplinari, field, impact |
| 4 | 6.656 | 4.187 | 7 | capac, research, polici, build, cultur |
| 5 | 6.142 | 3.566 | 2 | popul, level |
| 6 | 5.403 | 2.760 | 4 | poor, rural, prioriti, share |
| 7 | 4.564 | 1.969 | 4 | digit, skill, manag, connect |
| 8 | 4.179 | 1.651 | 4 | shortag, imped, low, mention |
| 9 | 3.773 | 1.346 | 2 | meat, devic |
| 10 | 3.694 | 1.289 | 1 | issu |

**Table S15. Latent Semantic Analysis-based topic modelling across the future opportunity dimension of one health discourse in the Global South. For each of the extracted topics, the percentage of explained variance, number of terms, and terms are reported.**

| **Topic** | **Eigenvalue** | **Explained Variance (%)** | **Number of Terms** | **Terms** |
| --- | --- | --- | --- | --- |
| 1 | 24.713 | 58.780 | 61 | health, diseas, anim, improv, environment |
| 2 | 7.353 | 5.203 | 17 | healthcar, respons, monitor, time, inform |
| 3 | 7.108 | 4.862 | 11 | food, environ, resist, understand, profession |
| 4 | 6.283 | 3.800 | 4 | system, develop, implement, expertis |
| 5 | 5.942 | 3.398 | 7 | conserv, secur, divers, potenti, domain |
| 6 | 5.679 | 3.104 | 4 | issu, infrastructur, record, electron |
| 7 | 5.125 | 2.528 | 1 | bring |
| 8 | 4.758 | 2.179 | 3 | care, popul, access |
| 9 | 4.684 | 2.112 | 10 | peopl, studi, encourag, awar, infecti |
| 10 | 4.456 | 1.911 | 6 | addit, futur, earli, medicin, model |

**Table S15. Latent Semantic Analysis-based topic modelling across the future challenge dimension of one health discourse in the Global South. For each of the extracted topics, the percentage of explained variance, number of terms, and terms are reported.**

| **Topic** | **Eigenvalue** | **Explained Variance (%)** | **Number of Terms** | **Representative Terms** |
| --- | --- | --- | --- | --- |
| 1 | 25.662 | 59.652 | 59 | health, challeng, resourc, environment, diseas |
| 2 | 9.287 | 7.813 | 14 | address, digit, manag, access, shortag |
| 3 | 7.774 | 5.474 | 14 | food, veterinari, framework, integr, promot |
| 4 | 7.104 | 4.571 | 3 | insuffici, relat, outbreak |
| 5 | 5.630 | 2.871 | 6 | initi, coordin, support, financi, regulatori |
| 6 | 5.210 | 2.459 | 10 | time, skill, data, local, level |
| 7 | 4.790 | 2.078 | 7 | opportun, studi, enabl, direct, environ |
| 8 | 4.515 | 1.847 | 5 | limit, cultur, lack, econom, slow |
| 9 | 4.231 | 1.621 | 6 | weak, collect, real, potenti, popul |
| 10 | 3.944 | 1.409 | 4 | emerg, agenc, ressourc, easi |

**Table S16. Latent Semantic Analysis-based topic modelling across the opportunity dimension of one health discourse in the Global South in female respondents. For each of the extracted topics, the percentage of explained variance, number of terms, and terms are reported.**

| **Topic** | **Eigenvalue** | **Explained Variance (%)** | **Number of Terms** | **Terms** |
| --- | --- | --- | --- | --- |
| 1 | 10.856 | 73.200 | 48 | health, diseas, environment, sustain, integr |
| 2 | 2.953 | 5.417 | 3 | ai, grow, popul |
| 3 | 2.826 | 4.961 | 4 | improv, respons, manag, prevent |
| 4 | 2.515 | 3.929 | 1 | Sector |
| 5 | 2.449 | 3.727 | 3 | peopl, live, care |
| 6 | 2.186 | 2.967 | 3 | treatment, applic, increas |
| 7 | 1.953 | 2.368 | 2 | work, smart |
| 8 | 1.408 | 1.231 | 1 | Awar |
| 9 | 1.353 | 1.137 | 1 | Servic |
| 10 | 0.995 | 0.615 | 56 | univers, remot, train, key, solut |

**Table S17. Latent Semantic Analysis-based topic modelling across the opportunity dimension of one health discourse in the Global South in male respondents. For each of the extracted topics, the percentage of explained variance, number of terms, and terms are reported.**

| **Topic** | **Eigenvalue** | **Explained Variance (%)** | **Number of Terms** | **Terms** |
| --- | --- | --- | --- | --- |
| 1 | 23.227 | 57.454 | 47 | health, human, diseas, anim, environment |
| 2 | 8.812 | 8.270 | 14 | predict, monitor, model, manag, smart |
| 3 | 8.192 | 7.146 | 8 | field, antimicrobi, resist, signific, sector |
| 4 | 7.222 | 5.554 | 11 | research, ai, polici, awar, complex |
| 5 | 6.050 | 3.898 | 10 | lead, biodivers, includ, knowledg, program |
| 6 | 5.369 | 3.070 | 7 | anthrax, control, invest, vaccin, relat |
| 7 | 4.573 | 2.227 | 7 | peopl, communiti, access, infecti, enabl |
| 8 | 4.184 | 1.864 | 9 | inform, record, electron, popul, remot |
| 9 | 3.875 | 1.599 | 4 | surveil, expert, risk, vector |
| 10 | 3.284 | 1.149 | 5 | resourc, care, share, increas, support |

**Table S18. Latent Semantic Analysis-based topic modelling across the challenge dimension of one health discourse in the Global South in female respondents. For each of the extracted topics, the percentage of explained variance, number of terms, and terms are reported.**

| **Topic** | **Eigenvalue** | **Explained Variance (%)** | **Number of Terms** | **Terms** |
| --- | --- | --- | --- | --- |
| 1 | 11.158 | 61.941 | 45 | health, environment, challeng, collabor, infrastructur |
| 2 | 5.433 | 14.683 | 10 | popul, practic, adopt, understand, level |
| 3 | 4.215 | 8.837 | 7 | issu, data, fund, insuffici, limit |
| 4 | 3.271 | 5.323 | 2 | build, anim |
| 5 | 2.177 | 2.358 | 2 | devic, research |
| 6 | 1.963 | 1.917 | 1 | poor |
| 7 | 1.775 | 1.568 | 1 | cross |
| 8 | 1.414 | 0.995 | 2 | emerg, econom |
| 9 | 1.414 | 0.995 | 2 | technolog, cultur |
| 10 | 1.352 | 0.910 | 36 | collect, meat, crucial, principl, region |

**Table S19. Latent Semantic Analysis-based topic modelling across the challenge dimension of one health discourse in the Global South in male respondents. For each of the extracted topics, the percentage of explained variance, number of terms, and terms are reported.**

| **Topic** | **Eigenvalue** | **Explained Variance (%)** | **Number of Terms** | **Terms** |
| --- | --- | --- | --- | --- |
| 1 | 23.749 | 65.811 | 57 | health, limit, environment, resourc, implement |
| 2 | 8.682 | 8.796 | 19 | surveil, econom, includ, chang, degrad |
| 3 | 6.727 | 5.281 | 9 | capac, research, cultur, build, respons |
| 4 | 5.680 | 3.764 | 3 | prioriti, share, area |
| 5 | 5.051 | 2.977 | 8 | poor, digit, skill, imped, rural |
| 6 | 4.353 | 2.211 | 3 | connect, low, personnel |
| 7 | 3.957 | 1.827 | 5 | communiti, meat, devic, popul, mention |
| 8 | 3.318 | 1.284 | 1 | lack |
| 9 | 3.114 | 1.131 | 2 | scienc, field |
| 10 | 2.943 | 1.011 | 1 | framework |

**Table S20. Latent Semantic Analysis-based topic modelling across the future opportunity dimension of one health discourse in the Global South in female respondents. For each of the extracted topics, the percentage of explained variance, number of terms, and terms are reported.**

| **Topic** | **Eigenvalue** | **Explained Variance (%)** | **Number of Terms** | **Terms** |
| --- | --- | --- | --- | --- |
| 1 | 11.803 | 68.632 | 47 | health, diseas, environment, opportun, integr |
| 2 | 6.392 | 20.125 | 20 | monitor, rapid, solut, coordin, addit |
| 3 | 2.434 | 2.917 | 6 | indigen, life, awar, promot, develop |
| 4 | 2.372 | 2.771 | 4 | increas, technolog, advanc, collabor |
| 5 | 1.709 | 1.439 | 1 | diagnost |
| 6 | 1.387 | 0.948 | 1 | reach |
| 7 | 0.990 | 0.482 | 1 | data |
| 8 | 0.000 | 0.000 | 44 | epidem, issu, antimicrobi, profession, futur |

**Table S21. Latent Semantic Analysis-based topic modelling across the future opportunity dimension of one health discourse in the Global South in male respondents. For each of the extracted topics, the percentage of explained variance, number of terms, and terms are reported.**

| **Topic** | **Eigenvalue** | **Explained Variance (%)** | **Number of Terms** | **Terms** |
| --- | --- | --- | --- | --- |
| 1 | 22.450 | 60.286 | 55 | health, diseas, anim, improv, human |
| 2 | 7.099 | 6.028 | 16 | healthcar, food, develop, resourc, sustain |
| 3 | 6.433 | 4.951 | 4 | system, expertis, implement, manag |
| 4 | 5.731 | 3.928 | 10 | issu, record, infrastructur, solut, outcom |
| 5 | 5.277 | 3.331 | 3 | inform, platform, bring |
| 6 | 5.015 | 3.008 | 10 | communiti, peopl, studi, time, respons |
| 7 | 4.742 | 2.690 | 3 | access, remot, epidem |
| 8 | 4.559 | 2.486 | 9 | earli, encourag, medicin, infecti, addit |
| 9 | 4.002 | 1.916 | 7 | model, mobil, diagnost, rapid, ebola |
| 10 | 3.663 | 1.605 | 7 | care, popul, level, electron, coordin |

**Table S22. Latent Semantic Analysis-based topic modelling across the future challenge dimension of one health discourse in the Global South in female respondents. For each of the extracted topics, the percentage of explained variance, number of terms, and terms are reported.**

| **Topic** | **Eigenvalue** | **Explained Variance (%)** | **Number of Terms** | **Representative Terms** |
| --- | --- | --- | --- | --- |
| 1 | 11.754 | 78.500 | 58 | health, challeng, infrastructur, environment, communiti |
| 2 | 3.821 | 8.295 | 5 | manag, regulatori, cultur, fund, polit |
| 3 | 3.372 | 6.459 | 9 | emerg, climat, diseas, tackl, ressourc |
| 4 | 1.959 | 2.180 | 2 | econom, popul |
| 5 | 1.566 | 1.394 | 2 | data, collect |
| 6 | 1.000 | 0.568 | 1 | real |
| 7 | 0.000 | 0.000 | 51 | disciplin, outbreak, region, opinion, environ |

**Table S23. Latent Semantic Analysis-based topic modelling across the future challenge dimension of one health discourse in the Global South in male respondents. For each of the extracted topics, the percentage of explained variance, number of terms, and terms are reported.**

| **Topic** | **Eigenvalue** | **Explained Variance (%)** | **Number of Terms** | **Representative Terms** |
| --- | --- | --- | --- | --- |
| 1 | 23.920 | 61.654 | 48 | health, resourc, challeng, anim, diseas |
| 2 | 9.224 | 9.169 | 28 | address, digit, build, south, global |
| 3 | 7.314 | 5.765 | 8 | food, improv, integr, outbreak, promot |
| 4 | 5.632 | 3.418 | 9 | coordin, support, financi, govern, regulatori |
| 5 | 5.093 | 2.795 | 7 | skill, level, local, data, respons |
| 6 | 4.814 | 2.497 | 9 | opportun, studi, enabl, direct, infecti |
| 7 | 4.447 | 2.131 | 3 | limit, cultur, econom |
| 8 | 4.320 | 2.011 | 6 | time, lack, partnership, real, tackl |
| 9 | 3.417 | 1.258 | 4 | care, easi, remot, insuffici |
| 10 | 3.280 | 1.159 | 6 | poor, low, network, popul, leadership |

**Table S24. Latent Semantic Analysis-based topic modelling of the convergence of one health and digital health discourse in the Global South. For each of the extracted topics, the percentage of explained variance, number of terms, and terms are reported.**

| **Topic** | **Eigenvalue** | **Explained Variance (%)** | **Number of Terms** | **Representative Terms** |
| --- | --- | --- | --- | --- |
| 1 | 35.846 | 75.232 | 86 | health, digit, data, diseas, approach |
| 2 | 9.626 | 5.425 | 20 | global, converg, sector, recognit, climat |
| 3 | 8.201 | 3.937 | 5 | fund, support, organ, pandem, holist |
| 4 | 7.178 | 3.016 | 8 | manag, risk, limit, ai, requir |
| 5 | 5.558 | 1.809 | 3 | domain, countri, deliveri |
| 6 | 5.049 | 1.493 | 3 | south, impact, multisector |
| 7 | 4.904 | 1.408 | 5 | ministri, intern, stakehold, lack, reli |
| 9 | 3.428 | 0.688 | 1 | awar |
| 10 | 3.174 | 0.590 | 1 | level |

**Table S25. Latent Semantic Analysis-based topic modelling of the convergence of one health and digital health discourse in the Global South in female respondents. For each of the extracted topics, the percentage of explained variance, number of terms, and terms are reported.**

| **Topic** | **Eigenvalue** | **Explained Variance (%)** | **Number of Terms** | **Representative Terms** |
| --- | --- | --- | --- | --- |
| 1 | 10.140 | 75.046 | 44 | health, approach, environment, collabor, communiti |
| 2 | 2.557 | 4.772 | 1 | manag |
| 3 | 2.481 | 4.492 | 5 | increas, impact, awar, climat, chang |
| 4 | 2.196 | 3.519 | 2 | share, ministri |
| 5 | 2.112 | 3.257 | 3 | fund, implement, initi |
| 6 | 1.995 | 2.904 | 3 | servic, effect, reach |
| 7 | 1.477 | 1.592 | 1 | public |
| 8 | 1.414 | 1.460 | 2 | avail, inform |
| 9 | 1.410 | 1.452 | 1 | key |
| 10 | 1.031 | 0.776 | 70 | understand, limit, underserv, effici, antimicrobi |

**Table S26. Latent Semantic Analysis-based topic modelling of the convergence of one health and digital health discourse in the Global South in male respondents. For each of the extracted topics, the percentage of explained variance, number of terms, and terms are reported.**

| **Topic** | **Eigenvalue** | **Explained Variance (%)** | **Number of Terms** | **Representative Terms** |
| --- | --- | --- | --- | --- |
| 1 | 34.851 | 77.313 | 87 | health, digit, data, diseas, human |
| 2 | 9.396 | 5.619 | 20 | global, sector, converg, recognit, climat |
| 3 | 8.136 | 4.214 | 5 | support, fund, organ, pandem, holist |
| 4 | 7.138 | 3.243 | 9 | manag, risk, limit, ai, requir |
| 5 | 5.061 | 1.630 | 3 | rise, south, multisector |
| 6 | 4.869 | 1.509 | 5 | ministri, intern, lack, reli, stakehold |
| 8 | 3.167 | 0.638 | 1 | level |
| 10 | 2.840 | 0.513 | 2 | countri, domain |
